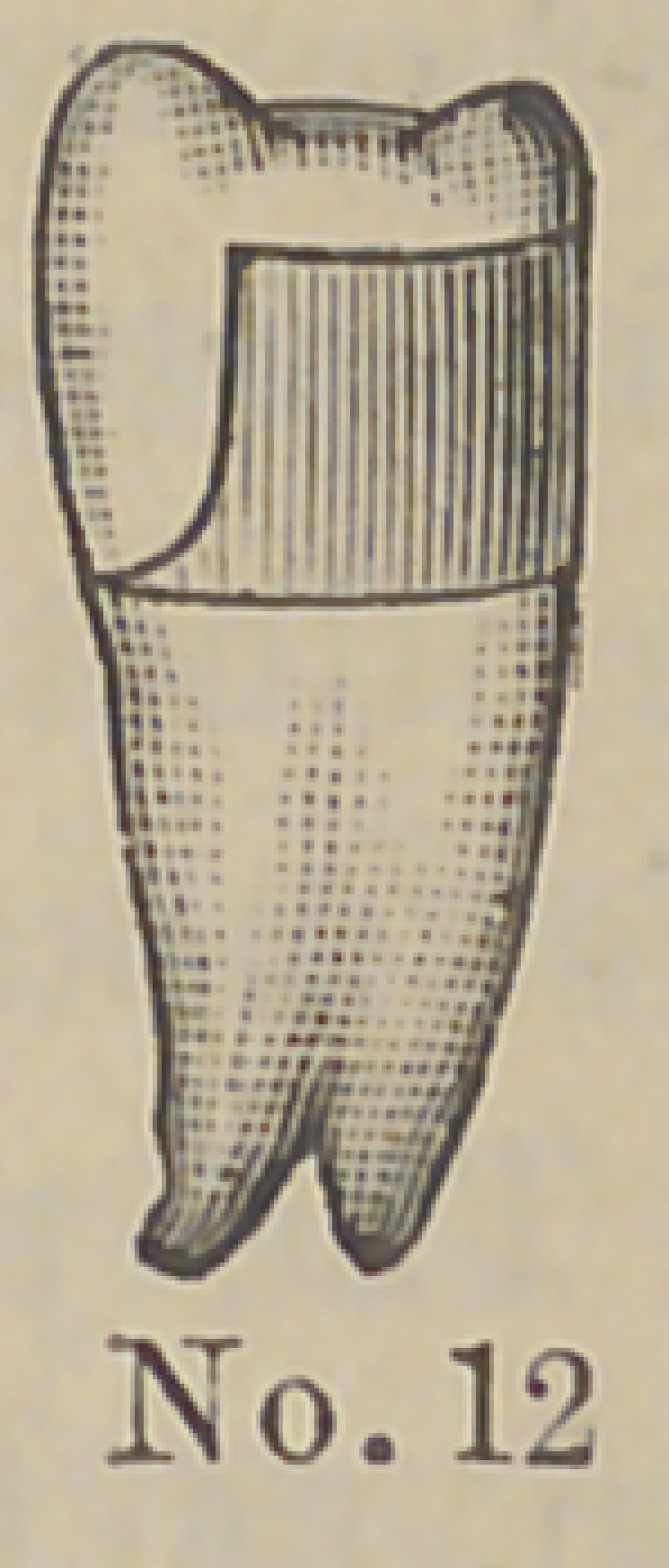# A Case in Hand

**Published:** 1890-07

**Authors:** G. E. Corbin

**Affiliations:** St. Johns, Mich.


### A Case in Hand.

BY G. E. CORBIN, M.D., D.D.S., ST. JOHNS, MICH.

The danger of contracting the disease when exposed to a well-marked case of small pox, has for many centuries been well understood. The contagiousness of measles and of scarlet fever has long been conceded. The possibility of transmitting tuberculosis from one person to another did not seem to arrest any general attention until within the last quarter of a century. In a general way, reason has, perhaps, always understood that each effect must have been preceded by a cause; but we are indebted to the combined efforts of microscopists and pathologists for the tracing, isolating, and exhibiting of particular disease germs, which, when planted in suitable soil, invariably produce like conditions of disease in any number of human organisms.

A specific bacillus has been isolated, cultivated, propagated and inoculated into the tissues of various inferior animals, invariably resulting in the production of tuberculosis. Cases are well authenticated where syphilis has been transmitted from one person to another by kissing, by smoking the same pipe, and by other innocent but untidy habits. Diphtheria has been transmitted from one person to another by drinking from the same goblet.

Such facts have caused conscien[illegible]s modern surgeons to be exceedingly particular in all their ope[illegible]ns about cleaning and sterilizing their instruments.

Should *dental* surgeons be less so? In November, 1877, I was called in consultation with the attending physician, and found a sallow, despondent, and somewhat emaciated patient who gave the following brief history: Three months previously she had the right inferior first molar extracted, the tooth all coming out at the first effort. She stoutly maintained that she noticed at the time that the beaks of the forceps were disgustingly soiled with dried blood ; but they were quickly used, and before she had summoned courage to remonstrate. At the time she felt only disgust for the foulness. At first there was no more pain or soreness than she expected but it did not cease or diminish.

In the course of a few weeks the pain became decidedly severe, and the flesh in and about the socket presented a raw and angry appearance. At this stage the patient conceived the idea that she had been poisoned by the foul forceps, an opinion she maintained until the day of her death. At the time the writer hereof first saw the patient pain, sleeplessness, anxiety, anorexia, etc., had not only produced emaciation, but a general cachectic appearance; the alveolus was thickened, and partially covered by a soft, brain-like, vascular tumor that protruded from the socket of the extracted tooth. Attempts had been made to destroy it by cauterization.

On such occasions the hemorrage was troublesome and the tumor was rapidly reproduced and increased in size. Liquid foods only could be taken, and so vascular and irritable was the excrescence that the efforts at deglutition, coughing, etc., were often followed by hemorrhage.

The diagnosis at this stage of the disease was clearly either a soft sarcoma or an encephaloid cancer. As the lymphatic glands in the axilla were already much affected at the time of the consultation it was decided that an attempt at complete extirpation of the disease by excision of the jaw would be futile. The treatment, therefore, was subsequently palliative only. The patient lingered in much distress and died on the March following and in a little less than nine months from the date of extraction.

But that was a case in the mouth.

The "case in hand" was in the palm of my own hand. In February, 1888, in excavating a cavity of decay in a pulpless tooth in the mouth of a young and apparently healthful person, by carelessness, I plunged the point of a small excavator, loaded with debris, into the palm of my left hand at just about the middle of the fourth metacarpal bone.

The wound produced was a small puncture just sufficiently deep to pierce the skin and not severe enough to cause the loss of a particle of blood. Indeed, as a matter of precaution, I tried to force a drop of blood from the wound by squeezing it, but failed. I thought of enlarging the opening and cauterizing the parts but did not do so. For a few days the trouble was scarcely noticeable, then for many weeks there was a soft, pulpy condition just beneath the skin surrounded by a slightly inflamed border.

At this stage a free incision seemed called for, but was daily postponed, waiting for the advent of expected suppuration. The larger lymphatic vessels by their redness and tenderness were easily traceable from the hand to the elbow, and the axillary glands were enlarged and tender. This condition lingered for weeks, causing much pain and considerable apprehension but no loss of time. Finally, instead of suppurating, the soft and pulpy condition gradually subsided. The inflamed condition of the lymphatic vessels and glands simultaneously subsided but the abnormal tissue at the seat of the injury as it became firmer in texture, also increased in quantity, until at the expiration of twenty-two months the palm of the hand was encumbered with a subcutaneous tumor about the size and shape of a common white bean. In December, 1889, I decided on its removal and not relishing the taunt that "a physician never takes his own medicine," I determined to do it myself.

As one hand only could be made available for manipulative purposes, the other being expected to maintain a very passive condition in the transaction, I set myself about devising and securing some necessary aids and appliances. For the purpose of keeping the lips of the wound properly asunder during the process of dissection I constructed from a piece of small clock spring, a spring with each end sharpened and so curved that when the ends were brought in contact and inserted between the lips of the wound, the spring was securely retained, and did its work perfectly. I drew the temper from an ordinary sewing needle of moderate size and curved it at the point to the shape of a small tenaculum. This was threaded with waxed floss silk with which it was secured to the end of my "ring finger," the extension of which was expected to produce the traction, through the tenaculum, on the tumor necessary to lift it from its bed during the process of dissection.

My only assistant was the companion who more than a quarter of a century before had promised to stand by me both in prosperity and affliction. Accordingly she seated herself in a chair in the opposite corner of my office, placed her elbows on her knees, and covered both eyes and both ears with her hands.

Thus fortified and sustained I proceeded. I first made a liberal application of a ten per cent. solution of cocaine which very soon blanched the parts. With a bistoury I laid the skin open for five-eighths of an inch and was gratified to find the cocaine had done excellent work. I then inserted the ends of the steel spring and filled the wound with cocaine but it produced no observable effect on the tissues beneath the skin.

I inserted the needle tenaculum into the tumor and waited for the fibers of the extensior digitorum communis to contract, and with a small tenotomy knife proceeded to trace out the line of junction between the normal and abnormal tissues. This proved to be decidedly more a work of necessity than of pleasure. The hemorrhage was considerable, and that, or something else caused frequent interruption and much delay, but I economized the seasons of delay in meditation. In fact, most of the hour consumed was occupied in meditation. I reflected that "I had done those things which I ought not to have done, and in this particular case, that I had left undone the very thing that I ought to have done"—*cauterization.*

Time, patience, and perseverance accomplished the work. The tumor out; with a surgeon's curved needle I took one deep stitch, bringing the edges of the wound closely in apposition, and by the aid of my teeth, which are not artificial, I succeeded in tying a secure knot.

I clipped the ends of the silk, covered all with adhesive plaster, met all my regular daily engagements at the dental chair, and now after a lapse of five months the difficulty seems to have been completely removed.

---

After the reading of Dr. Corbin's paper, the Association adjourned until 2 P.M.

WEDNESDAY, 2 P.M.

The afternoon was given up to clinics.

Dr. W. B. Ames, of Chicago, showed a method of manipulating copper amalgam. He takes his extra dry amalgam and holding a piece with the foil carries in the flame of the lamp until the slight bubbles of mercury appear on the surface, it is then put in a wedgwood mortar and crushed to a dry powder; then add nitrate of mercury and triturate until the mass becomes pasty and of proper condition to use in filling; wash to remove acid and proceed to use in filling. The doctor prefers to heat the amalgam in the pliers rather than the iron spoon for the reason that it is more uniformly heated. The nitrate of mercury is made by the action of dilute nitric acid—1 part acid and 5 parts water—on mercury. The mercury is put into the bottle and the acid poured over it and allowed to stand several days until no bubbles are seen to rise in the fluid from the action on the mercury. The claim is that the nitrate of mercury when triturated in the mortar with the powdered amalgam, gives up sufficient of its mercury to render the powder plastic and suitable for inserting into the tooth, and in this way only so much mercury as is necessary is used. Dr. Ames also claims that there will be no wasting away of copper amalgam if prepared in this manner.

Dr. Case, of Jackson, demonstrated a method of swedging caps for all gold crowns and caps for bridge teeth. The method is to imbed a natural tooth in a section of gas pipe about an inch long and three-fourths of an inch in diameter, with moldine or plaster of paris so that only the crown surface and so much of the lateral surfaces as may be desirable are exposed; a piece of rubber tubing is then placed over the cylinder containing the tooth so that it extends one half inch, or higher if desired, above the crown; into this is poured Babbits metal or any fusible alloy that is sufficiently hard to endure the swedging without changing form. This secures a matrix mould of the natural crown. To swedge the gold cap a piece of gold of required size and thickness is laid over this matrix and driven into it by a piece of lead. The method is very similar to swedging caps on the ordinary die plate, but it has the advantage of securing any sized cap that may be needed and a greater variety of shapes. Of course it involves keeping near at hand a large assortment of teeth with which the matrix may be made.

Dr. B. S. Palmer, of Chicago, exhibited a pneumatic mallet, which is attached to and operated by the motive power of the dental engine. The action is secured by a piston attached by an eccentric to the rod of the dental engine, working in a cylinder which is secured to the standard of the engine. By the action of this piston in the cylinder, the air is alternately exhausted and forced into the air chamber of the hand-piece by means of rubber tubing connecting them. To change the action of the plugger to the dental engine hand-piece requires only a passing of a finger behind the belt, which can be done while the engine is in motion. The advantage claimed over other pneumatics is rapidity of action and ease of manipulation.

Dr. J. E. Low, of Chicago,demonstrated his method of setting two new forms of crowns and pins for attaching them. The pins are made of aluminum and the accompanying cuts represent their form and the method of using them. The root canal and end of the root are cut out and trimmed by the well known instruments of Dr. Low.

No. 1 No. 2 No. 3 No. 4 No. 5

No. 8 No. 9 No. 10 No. 11 No. 12

The above cuts fairly illustrate the method and form of crowns. No. 1 illustrates a root trimmed and drilled out with the Low root trimmer and a thread cut in the upper part with a screw tap illustrated in No. 6. No. 2 illustrates the post or dowel pin, which is made of illuminum of different sizes to suit different sized roots. They are also made of platinum for cases where it is desirable to solder on a crown. No. 3 illustrates the post screwed into place by means of the carrier, No. 7. Cement or gutta percha can be used to seal the joint. No. 4 illustrates the crown and the manner of its attachment. The crown is concaved to fit the cap and is attached by means of cement. No. 5 represents a completed operation. The advantages claimed are durability as compared with other crowns of this class; easily and quickly applied, natural appearance and inexpensiveness. The apparent weak point in this crown is the cement joint between the post and porcelain

No. 6

No. 7

crown, but the close adaptation and the mechanical construction are such as to make a very strong and durable joint. Cuts Nos. 8 and 9 represent a gold socket to be attached in any way desired by the operator to bicuspid and molar roots. It can be soldered to a pin, attached by a collar or screw and cement. Cut No. 10 represents a porcelain crown that is set into this socket by cement. Cuts Nos. 11 and 12 illustrate the idea further. The advantage of this crown is that an all porcelain front is secured with a strong backing of gold, little or none of which is visible on the outside. This is particularly a valuable crown for use in making bridge work, as in case of an accident a new crown can readily be inserted without removing the bridge from the mouth.

Dr. Case illustrated with models a new method of making a cast Watt's metal plate, with rubber attachment, for lower dentures. The plaster cast with the teeth ground and waxed into position, as for an ordinary rubber case, are invested in one side of a Watt's metal flask. The upper section of a rubber flask is then placed on this and poured full of plaster. After the plaster has set the section of the rubber flask is parted from the Watt's metal section bringing with it the teeth, but leaving the wax that had held them in position in the Watt's metal section. This wax is then carved so as to make a mould for the cast metal plate. The upper section of the Watt's metal flask is then placed in position and filled with plaster. When set, the flask is separated, wax removed, dried and poured. This metal plate is then trimmed and placed in the rubber flask on the teeth; its place will easily be determined, as the impression of the wax base plate will be indicated in the plaster. The other side of the rubber flask is then placed over this and filled with plaster. After the plaster has set the flask is opened, packed with rubber and vulcanized as any ordinary rubber case.

Dr. E. C. Moore, of Detroit, showed a device for holding operating instruments, in the drawers of the bracket or cabinet case. The device consists of two metal plates, which have been stamped out with a die into grooves, so that when two of the plates are fastened together with rivets the grooves form a separate compartment large enough to contain a single instrument. This holder is held in place in the drawer by a screw passed through each side of the drawer into a holder where it fits so loosely as to permit the holder to be placed in any position desirable. The drawer has no bottom, and when it is pulled out of the case or bracket the holder immediately assumes a vertical position. One hand only is required to place the instrument and holder in a horizontal position and to shove the drawer into the table. As many of these holders as the depth of the drawer will contain can be put in each drawer. Two holders can be placed in the ordinary drawers of an Allen table, each holder containing from nine to twelve instruments. This device provides for such a classification of instruments as will be appreciated by every busy and methodical operator.

Dr. Knapp, of Jackson, showed a device for holding the rubber dam out of the way, and napkins in place in operations about the mouth; it also supported a magnifying glass and a reflector. The apparatus consists of a metallic plate swedged to fit the chin, and held in place by a strap passing around the head. The mouth mirror, or reflector, and magnifying glass are each supported by an arm consisting of a series of ball and socket joints attached to the face plate, permitting easy adjustment to any desired position.

WEDNESDAY EVENING SESSION.

Meeting called to order at 8:30 P. M. Minutes of previous meeting read and approved.

Dr. Dorrance, for the committee, read the following report on the death of Dr. Dyer:

WHEREAS, since the last meeting of this association our brother and Vice-President, Dr. C. H. Dyer, has been called away from the busy scenes of earth, and his activities in the dental profession have ceased forever, Therefore, be it *Resolved*, That this association desires to express the sense of loss it feels in his early demise, not only as an active member of the association and profession, but as a citizen, acquaintance and friend. For many years past Dr. Dyer has been actively identified with the progressive spirit of our vocation, and by careful study and constant endeavor to be as good as the best, he had become one of our foremost operators, and thoroughly posted in his calling.

Last year when the association met in the second city his efforts were untiring to make the meeting a success, and render our visit one of pleasure as well as profit. His social qualities were of the highest order, a fact which has given him a host of warm friends and genial companions.

And be it further *Resolved*, that a copy of this resolution be incorporated in the minutes of this meeting of the Association, and a copy also be sent to the family of the deceased brother, and to the journals for publication. Signed, W. H. Dorrance, L. D. Wood, and F. S. Owen.

The resolution was adopted by a rising vote.

The committee reported the following resolution in regard to the Dental Protective Association :

*Resolved*, That the Michigan Dental Association heartily approve the aim and plan of the Dental Protective Association, and that it is further

*Resolved*, That we deem it the duty of every member of the dental profession in the State of Michigan to join the Dental Protective Association, and further be it

*Resolved*, That Dr. Crouse is hereby requested to furnish an abstract of his remarks for publication in the dental journals. Signed by committee. On motion, the resolution was unanimously adopted.

The president declared the next order of business to be the discussion of Dr. Corbin's paper.

Dr. Dorrance said it was clear from the paper that all the trouble in these two cases had been caused by uncleanliness. Too little attention is given to cleaning dental instruments. All instruments should be washed and disinfected in a weak solution of carbolic acid or oil of eucalyptus.

Dr. J. Taft: Instances of inoculation by careless use of operating instruments are quite common. It requires only a slight amount of septic matter to produce serious consequences if the conditions are favorable. As an evidence of this we have only to step into the laboratory of our bacteriologists and see them take upon a needle point a slight amount of virus or disease germs and plant it in a specially prepared medium which is kept in favorable condition for only a short time when a large growth of the germs will result. And so it is when there is a low vitality of the oral tissues that a small wound with an instrument charged with only a slight amount of septic matter may produce very serious results. All instruments should be carefully washed and disinfected by dipping in a five to ten per cent. solution of carbolic acid. There are other good disinfectants, but none so effective, and non-injurious to polished instruments.

Another very disgusting habit is the use of rubber dam over and over again, as long as there is any possible chance to use it. Rubber dam is not so expensive an article as to justify such a practice in any case. Soiled napkins are also used in a similar way regardless of all sense of propriety or decency. There are many such practices that are not only indecent but are habits to do incalculable mischief.

Dr. Douglas: I think there is more danger to be expected from using instruments employed in removing tartar from teeth, especially in cases of pyorrhœa alveolaris. I always wash my rubber dam in a running stream of clean water and do not hesitate to use it again for another patient.

Dr. Brophy: I can heartily indorse Dr. Taft's remarks on cleanliness. I am certain that many cases of alveolar abscess have been caused by using broaches, for removing pulps, that have been used in the treatment of badly diseased teeth. It is my practice to tear my piece of rubber dam from the piece, use it but once, then throw it away, and I do the same in regard to napkins. I use antiseptic napkins prepared by Seabury & Johnson, and when through with them throw them into the waste basket. Too much care can not be exercised in regard to cleanliness of all kinds of dental instruments.

Dr. Field: I sometimes think cleanliness is almost a little better than Godliness. I cut my rubber dam six by nine inches, and after using it I have it carefully washed and put in an envelope with the patient's name on it, and when the patient returns for another sitting, I use the same piece again. My patients know this and appreciate it. I think it a good plan to let our patients know that we take precautions to be clean; make a little splashing when washing your hands so the patient will know you have washed your hands. I use my napkins over and over again—of course never the second time without washing—and like them better than new ones; they are softer and pleasanter to use. I have them washed at home, and they are thoroughly boiled in soap and water, and I believe they are as clean as though just bought out of the shop.

Dr. Crouse: I don't altogether agree with the author of the paper. I used to begin with a piece of rubber dam a yard square. The advantage of this is that it keeps my patient warm, protects his clothes from injury, and holds itself out of my way, and the saliva can not run in over it. I wash it again and again, and each time it seems softer and more agreeable to use. I feel sure that I can wash it perfectly clean. I don't think it is necessary to deceive my patients by "splashing the water when washing my hands" or pretending to be more cleanly than I really am. I endeavor to deal honestly with every patient that comes into my office, and try to make them feel that they can trust me. I don't throw away my tumblers because some one with a dirty mouth happens to drink out of one of them; neither do you refuse to drink at a public bar because every old toper in the country has drunk from the same glass before you. I believe rubber dam can be as thoroughly cleansed as your china ware. I don't want to bother with a little four by six piece of rubber dam. I most certainly believe that diseases may be disseminated by filthy practices, and by using infected instruments and appliances about the mouth, but at the same time I think there is such a thing as going too far with this antiseptic notion.

Dr. Scott, of Vicksburg: I want to make a public apology to Dr. Dorrance for criticising the use of modeling compound in taking impressions. I did not refer in particular to Dr. Dorrance, but thought there was a possibility of inoculating persons by using modeling compound that had previously been used in sore mouths for taking impressions.

Dr. Corbin was gratified that his paper had called out so much valuable discussion. He was of the opinion that the reason why so few punctured wounds were infected was that there was generally very free hemorrhage.

## Figures and Tables

**Figure f1:**
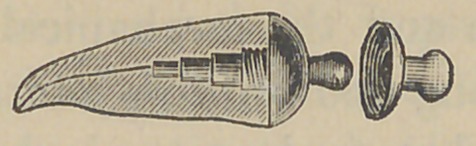


**No. 1. f2:**
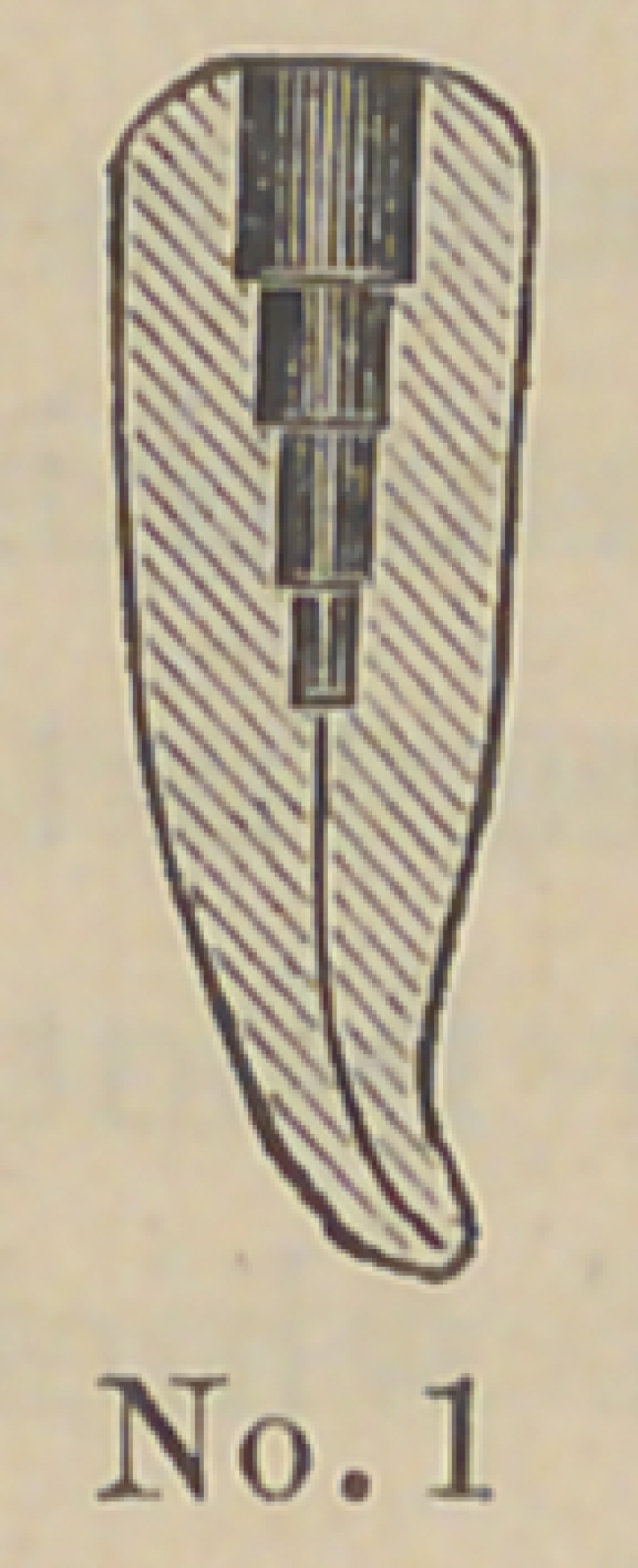


**No. 2. f3:**
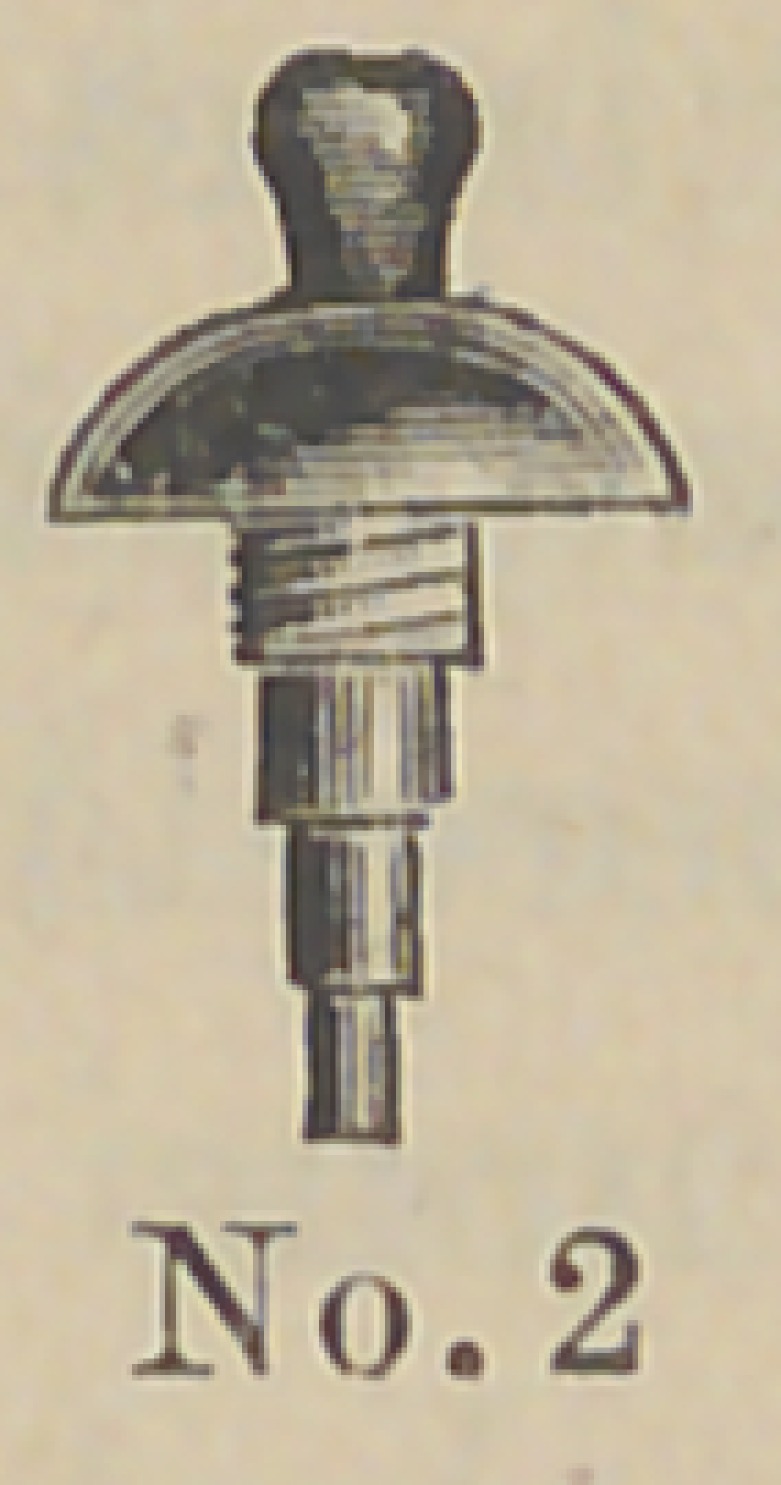


**No. 3. f4:**
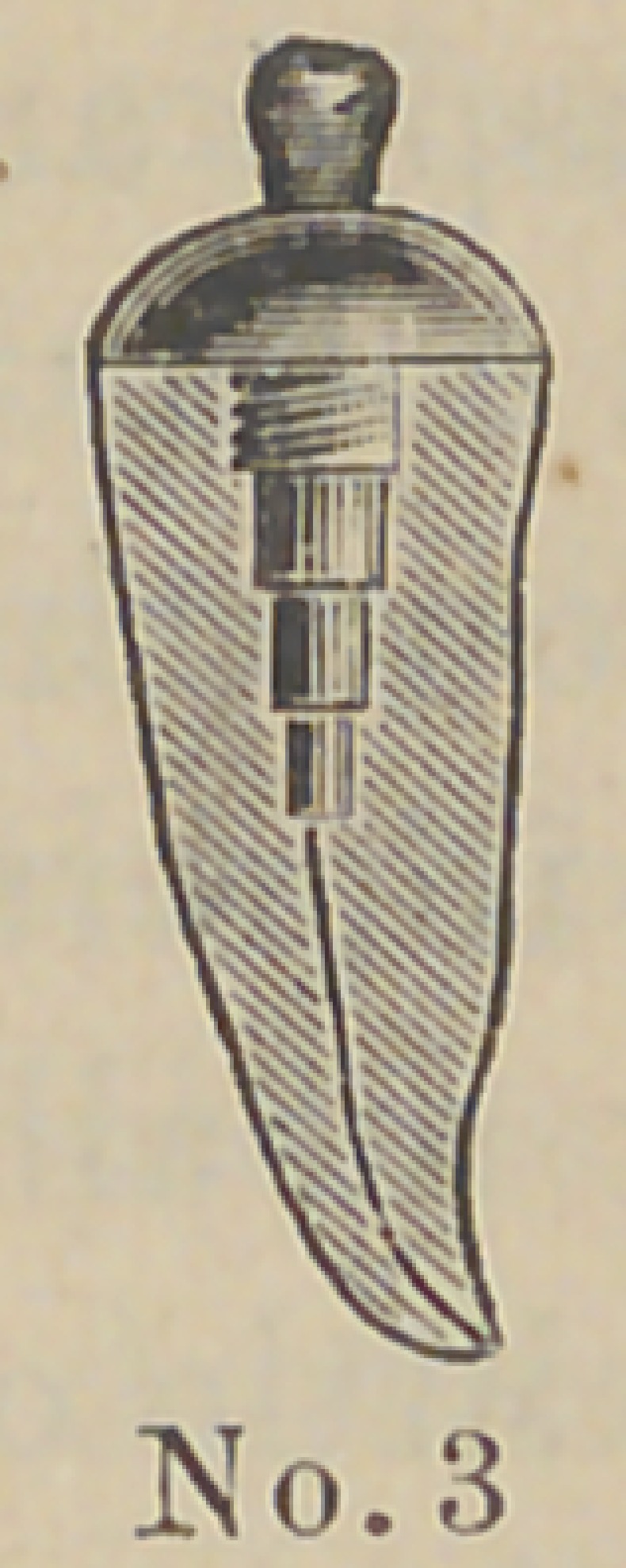


**No. 4. f5:**
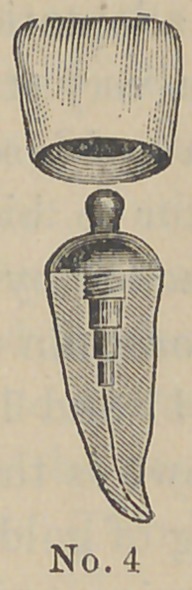


**No. 5. f6:**
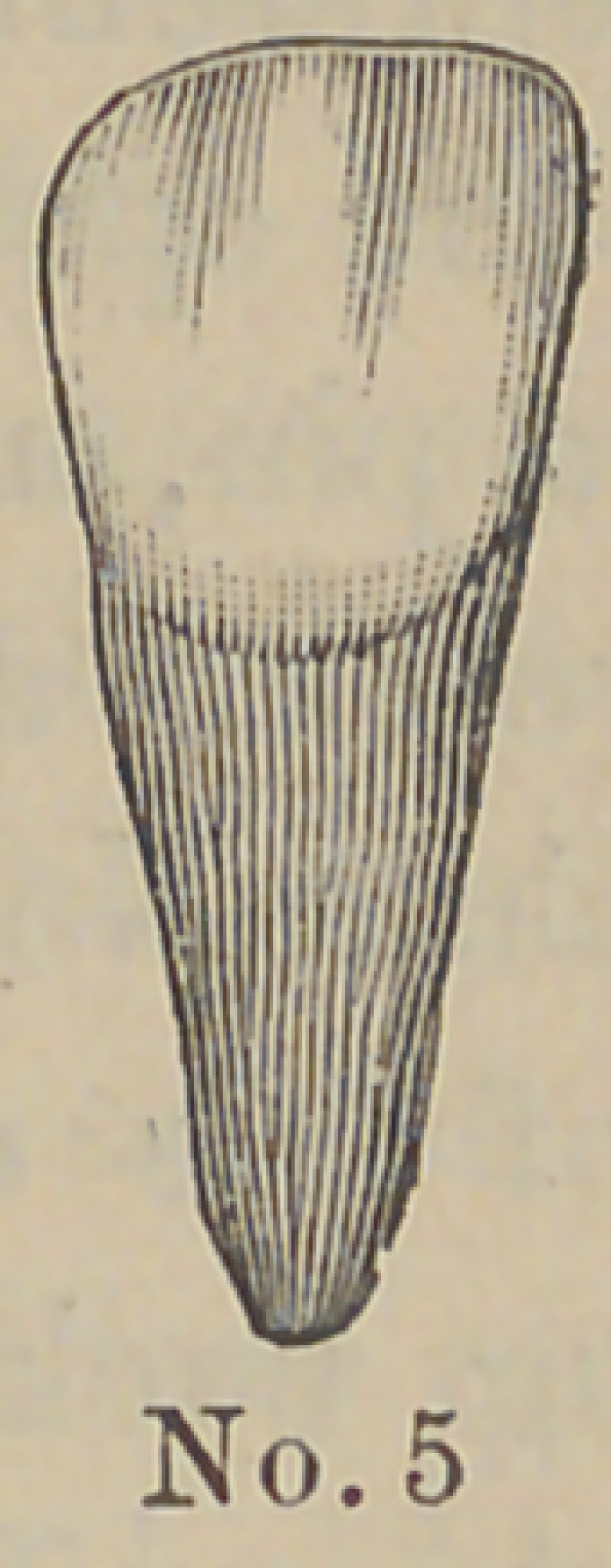


**No. 6. f7:**
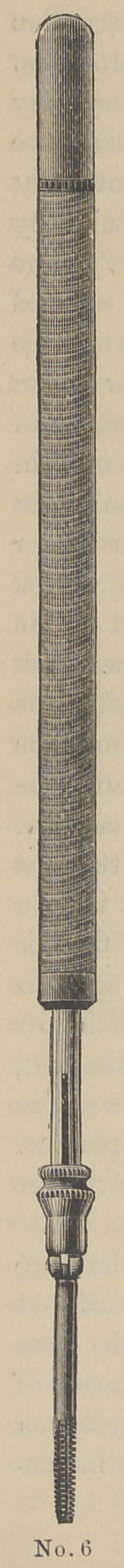


**No. 7. f8:**
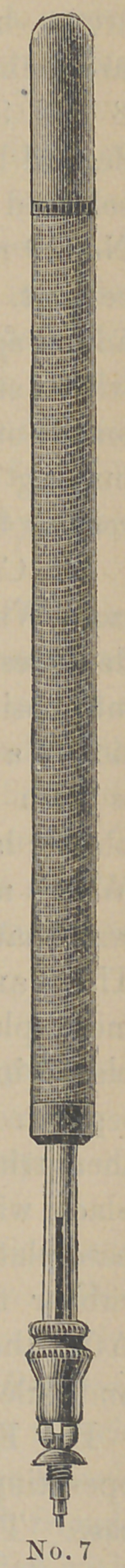


**No. 8. f9:**
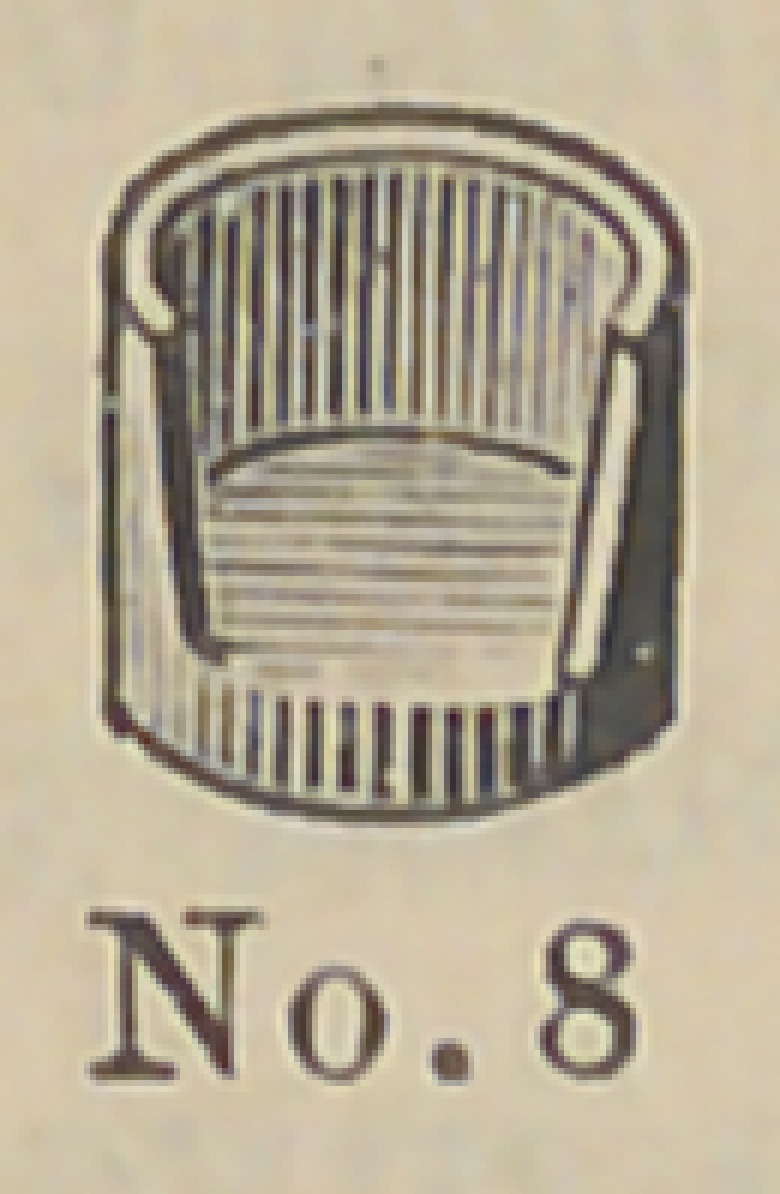


**No. 9. f10:**
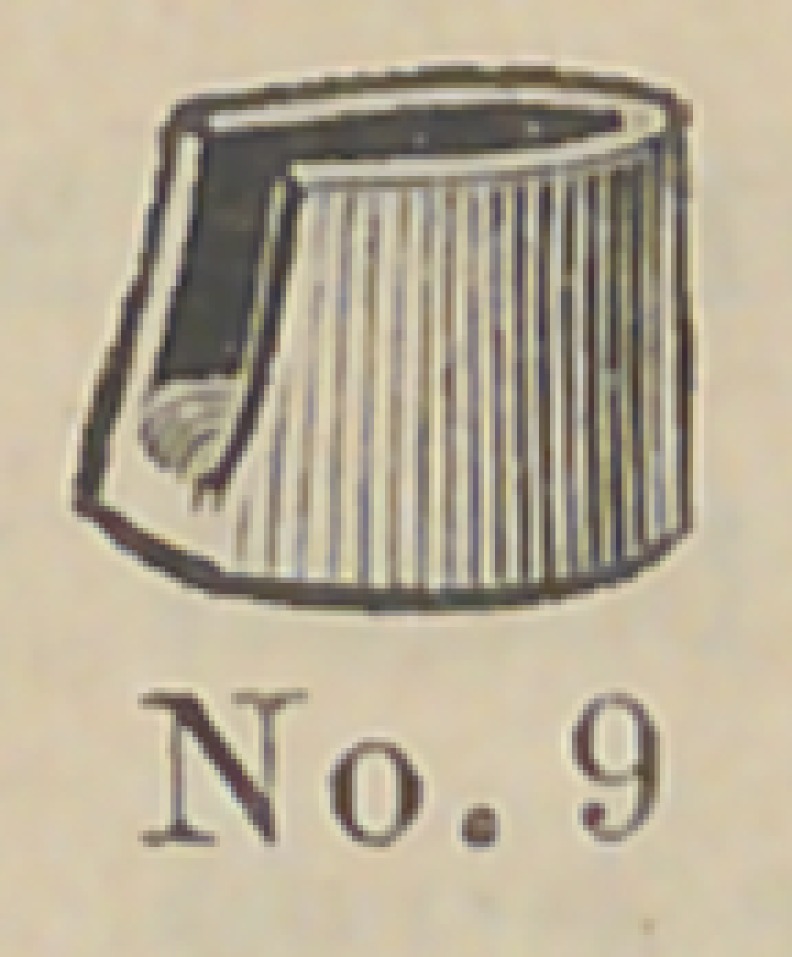


**No. 10. f11:**
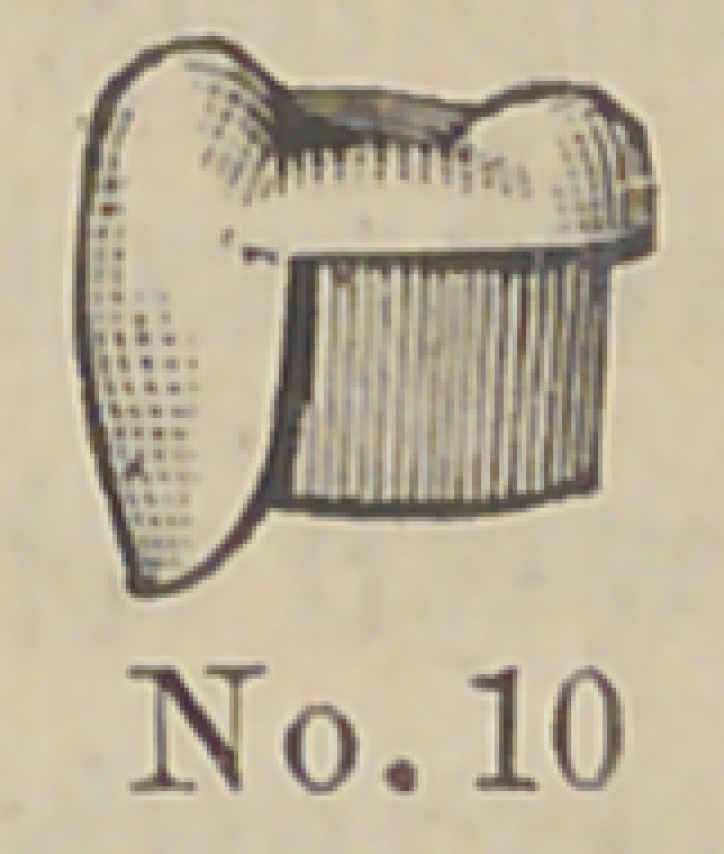


**No. 11. f12:**
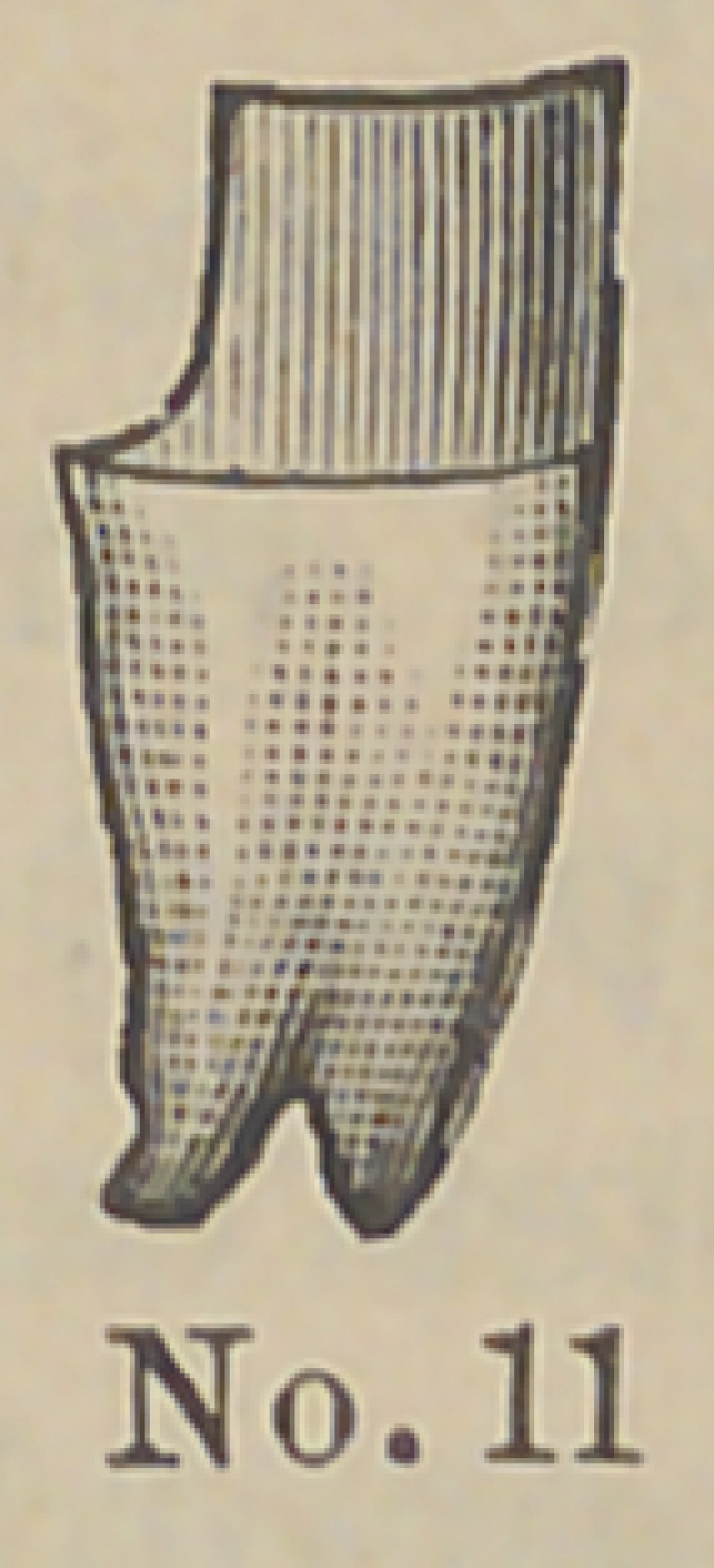


**No. 12. f13:**